# Solving the gender paradox in adolescent suicide: challenges and directions

**DOI:** 10.3389/fpsyt.2024.1386153

**Published:** 2024-06-19

**Authors:** Aaron Shengting Mai, Yi-Min Wan, Brendan Jen-Wei Tan, Eng-King Tan

**Affiliations:** ^1^ Department of Neurology, National Neuroscience Institute, Singapore, Singapore; ^2^ Yong Loo Lin School of Medicine, National University of Singapore, Singapore, Singapore; ^3^ Department of Psychiatry, Ng Teng Fong General Hospital, Singapore, Singapore; ^4^ Neuroscience and Behavioural Disorders, Duke-National University of Singapore (NUS) Medical School, Singapore, Singapore

**Keywords:** neurobiologic basis, suicide, genomics, epigenomics and epigenetics, adolescence

## Main text

The gender paradox in suicide has been described as early as 1998 (1), referring to the observation that while women demonstrated higher rates of suicide attempts, men had higher rates of death by suicide. This has prompted important epidemiological research into the gender differences in suicide, leading to consistent evidence supporting this observation. However, the biological basis for such gender differences is poorly understood, and there is increasing interest in the relationship between suicide, gender, and the underlying neurobiology. There is a pressing need to better understand both the psychosocial and neurobiological pathways of suicide, especially given the rise in suicide rates across all age groups in recent years.

**Figure 1:**
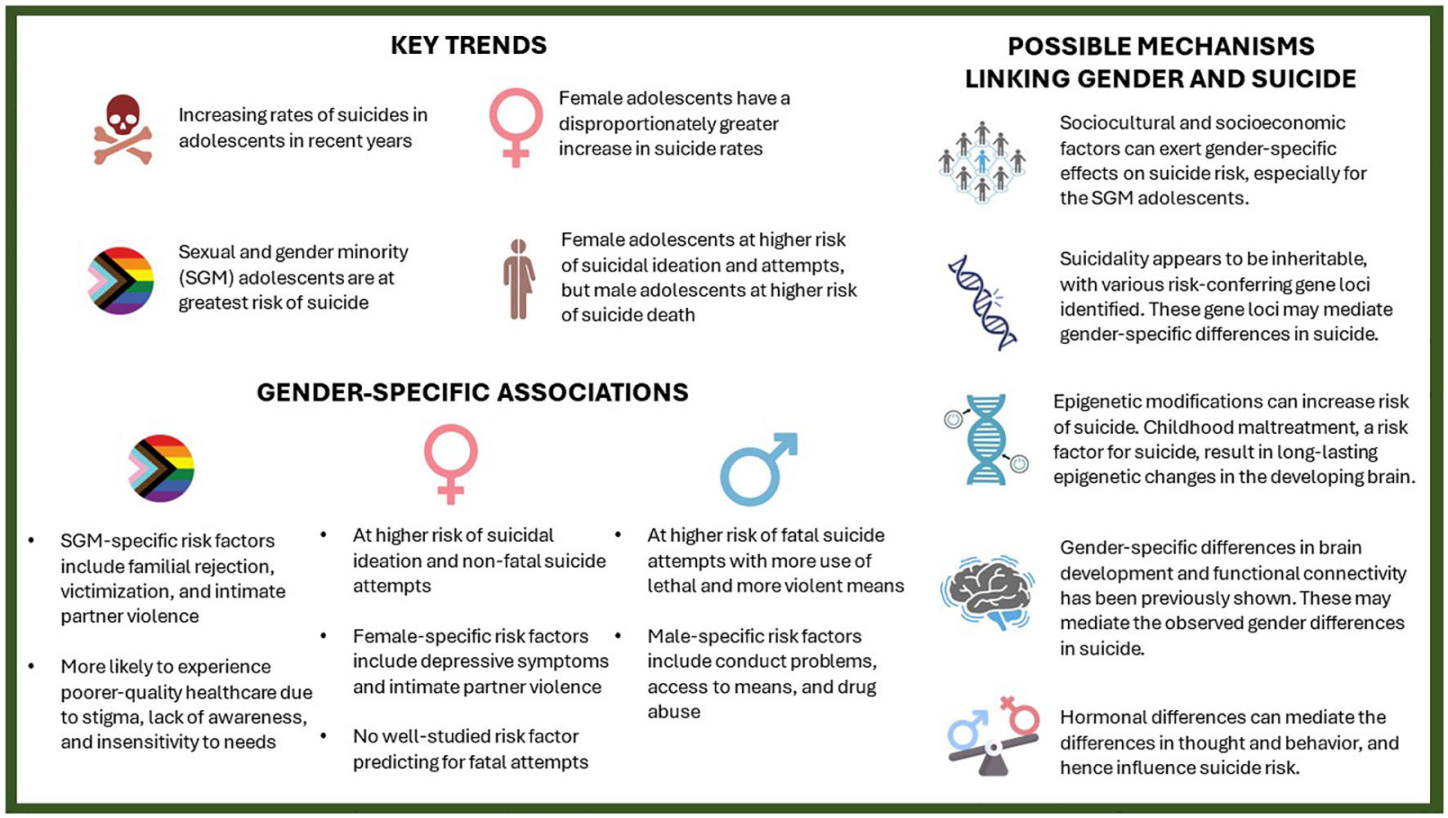
Key trends, gender-specific associations and their possible mechanisms in adolescent suicide.

This gender paradox is similarly observed in adolescents, who constitute an especially important demographic for suicide research, with suicide being a leading cause of death in this age group. Current suicide research has shown interesting but important gender-related differences in risk factors (**Figure 1**). Depressive symptoms and intimate partner violence are more important in female than male adolescents, the latter group is more affected by conduct problems, access to means, and drug abuse (2). Nonsuicidal self-injury, an important predictor of suicidality, similarly exhibits gender-related differences in terms of clinical characteristics and the suicide risk it confers (3).

That said, despite finding various predictors of suicide attempts in female adolescents, identifying risk factors predicting suicide completion in this subgroup remains a major challenge (2). While adolescent suicide rates are increasing for both genders, female adolescents experienced a disproportionate increase (4). The factors driving this observation remain unclear and are a topic of current debate. Answering this question will require a multidisciplinary approach, involving both national and international consortia, to evaluate and analyze the sociocultural, socioeconomic, and medical factors in adolescents across various countries.

Among adolescents, those of sexual and gender minorities (SGM) are at a higher risk of suicide, and they represent a particularly underserved and understudied population. Numerous studies have demonstrated that SGM adolescents, compared with their cisheterosexual counterparts, were more often subject to adverse childhood experiences (ACEs) (5). These include physical, emotional, and sexual abuse by parents, as well as being forced to conform to cisheteronormative standards and bullying by peers (5). Gender minority adolescents are most frequently affected by such ACEs, even when compared with sexual minority adolescents (5). Such ACEs can have a profound effect on their neurodevelopment and alter the biology of the brain, immune system, and the hypothalamus-pituitary-adrenal (HPA) axis (6–8), potentially explaining the exceptionally elevated suicide rates in gender minority adolescents. Sexual minority adolescents are also at higher risk of developing depressive symptoms compared with their heterosexual counterparts, starting as early as 11 years of age (9). The study’s authors found that disparities in depressive symptoms according to sexual orientation were more pronounced in girls than in boys, which they hypothesized to be a result of increased affiliative needs in girls. Lastly, SGM adolescents are more likely to experience a poorer quality of healthcare, which can be attributed to stigma, lack of awareness, and insensitivity to needs. Like female adolescents, evaluating the etiologic links underpinning the high association between SGM and suicide risk should be both a clinical and research priority.

Genetics and epigenetics could provide useful insight into the gender-based differences in suicide. Certain gene variants can intrinsically increase susceptibility to depression and suicidality through effects on neurochemistry (10), or interact with ACEs and stressful life events to result in suicidal behavior, in a gender-specific manner (7). Conversely, childhood adversities can also cause long-lasting gender-dependent epigenetic modifications both across the whole genome and specifically in the brain (6). As such, investigating gender differences in the common and rare gene variants linked to suicide, postmortem whole genome sequencing analysis of the brains in suicide deaths, and integrating them with brain-regulatory expression quantitative trait loci data will not only identify novel loci/genes but also help decipher their functional significance. Genetic and epigenetic epidemiology will be important in understanding adolescent suicide and explaining the clinically observed risk factors. Developing experimental gender-based animal “suicide” models may also unravel new pathophysiologic clues.

Neuroinflammation plays an important role in the etiopathogenesis of suicide. Higher levels of proinflammatory cytokines were observed in both suicide attempters and completers, with activation of the HPA axis and the kynurenine pathway. There is decreased production of brain-derived neurotrophic factor (BDNF) as a result, which is crucial in the modulation of neurotransmitters and neuronal plasticity. While there are limited studies on the gender-based effects on neuroinflammation, there is some evidence that at least BDNF demonstrates some gender-specific differences in adult suicide completers with depression (11). Analyzing the role of neuroinflammation in adolescents and its interaction with gender will yield helpful clues in understanding the gender paradox. Additionally, such research could also derive biomarkers for suicide risk, an idea that has been previously proposed, though their clinical utility remains to be validated.

To unravel the gender paradox puzzle among adolescents, the interactions of geographical area and specific sociocultural factors with other known suicidal risk factors should first be analyzed. Next, future studies ought to utilize comprehensive community- and family-based evaluation approaches, and systematically study potential neuroimaging and peripheral biomarkers that can predict different suicide behaviors (i.e., suicide ideation versus attempts versus completion). For example, previous research has found abnormalities in the hypothalamic-pituitary-adrenal (HPA) axis and serotonergic systems in suicide completers (12), as well as differences in prefrontal circuitry between suicide attempters and nonsuicidal patients were discovered on brain imaging (13, 14). Investigating how the stress response system (HPA axis) behaves differentially, the evolution of a growing brain in adapting to the various stressors, and the identification of authentic biomarkers that can predict suicidal behaviors can help provide further pathophysiologic clues to the intriguing gender paradox puzzle in adolescents.

Developing gender-based animal models, such as those investigating sexual dimorphisms in animal models of major depressive disorder (15), might also prove useful in investigating the relationship of biological sex and suicide. Most important is the sexual dimorphism in terms of neurochemistry, neurodevelopment (especially in the context of stresses in early life), and behavioral traits including impulsivity and aggression. Such models could potentially investigate the differences in phenotype of male versus female animal subjects when harboring the same genetic alterations or epigenetic modifications. While animal models cannot fully reproduce human conditions, comparative studies of male and female animal models can provide new pathophysiologic insights in terms of neural circuitry and humoral responses. Differences in genetics, epigenetics, and observed phenotypes can also result in varying response to certain treatments and management strategies (16). Such evidence would potentially prove useful in identifying at-risk individuals and guide treatment strategies.

In summary, the mechanisms driving the gender paradox in adolescent suicide remain to be further interrogated. Suicide risk arise from complex interactions from neurobiological factors (such as neuroendocrine, neurochemical, and inflammation) with events during development (such as ACEs and being of SGM) or in later life (such as stressful life events such as divorce, loss of loved ones, and sudden unemployment). Multi-omics and neuroimaging studies of suicide will be an important tool in studying the pathophysiology of the gender paradox and uncovering useful biomarkers. Developing gender-based animal models could assist us in understanding gender differences in traits linked with suicide, such as aggression and impulsivity. Understanding the gender paradox will guide the development and implementation of appropriate and cost-effective interventions for preventing adolescent suicide.

## Author contributions

AM: Conceptualization, Project administration, Visualization, Writing – original draft, Writing – review & editing. Y-MW: Writing – original draft, Writing – review & editing. BJ-WT: Writing – original draft, Writing – review & editing. E-KT: Conceptualization, Funding acquisition, Methodology, Project administration, Supervision, Validation, Writing – original draft, Writing – review & editing.
